# A key role for peroxynitrite-mediated inhibition of cardiac ERG (Kv11.1) K^+^ channels in carbon monoxide–induced proarrhythmic early afterdepolarizations

**DOI:** 10.1096/fj.201700259R

**Published:** 2017-07-25

**Authors:** Moza M. Al-Owais, Nishani T. Hettiarachchi, Hannah M. Kirton, Matthew E. Hardy, John P. Boyle, Jason L. Scragg, Derek S. Steele, Chris Peers

**Affiliations:** * Division of Cardiovascular and Diabetes Research, Leeds Institute of Cardiovascular and Metabolic Medicine, Faculty of Medicine and Health, University of Leeds, Leeds, United Kingdom; and; † Faculty of Biological Sciences, University of Leeds, Leeds, United Kingdom

**Keywords:** nitric oxide, arrhythmia, potassium channel

## Abstract

Exposure to CO causes early afterdepolarization arrhythmias. Previous studies in rats have indicated that arrhythmias arose as a result of augmentation of the late Na^+^ current. The purpose of the present study was to examine the basis for CO-induced arrhythmias in guinea pig myocytes in which action potentials (APs) more closely resemble those of human myocytes. Whole-cell current- and voltage-clamp recordings were made from isolated guinea pig myocytes as well as from human embryonic kidney 293 (HEK293) cells that express wild-type or a C723S mutant form of ether-a-go-go–related gene (ERG; Kv11.1). We also monitored the formation of peroxynitrite (ONOO^−^) in HEK293 cells fluorimetrically. CO—applied as the CO-releasing molecule, CORM-2—prolonged the APs and induced early afterdepolarizations in guinea pig myocytes. In HEK293 cells, CO inhibited wild-type, but not C723S mutant, Kv11.1 K^+^ currents. Inhibition was prevented by an antioxidant, mitochondrial inhibitors, or inhibition of NO formation. CO also raised ONOO^−^ levels, an effect that was reversed by the ONOO^−^ scavenger, FeTPPS [5,10,15,20-tetrakis-(4-sulfonatophenyl)-porphyrinato-iron(III)], which also prevented the CO inhibition of Kv11.1 currents and abolished the effects of CO on Kv11.1 tail currents and APs in guinea pig myocytes. Our data suggest that CO induces arrhythmias in guinea pig cardiac myocytes *via* the ONOO^−^-mediated inhibition of Kv11.1 K^+^ channels.—Al-Owais, M. M., Hettiarachchi, N. T., Kirton, H. M., Hardy, M. E., Boyle, J. P., Scragg, J. L., Steele, D. S., Peers, C. A key role for peroxynitrite-mediated inhibition of cardiac ERG (Kv11.1) K^+^ channels in carbon monoxide–induced proarrhythmic early afterdepolarizations.

Endogenous CO production occurs *via* degradation of heme by heme oxygenase (HO)-1 and -2 to provide protection from cellular stresses (1, 2). Endogenous cardiac CO provides cardioprotection, which limits the cellular damage of ischemia/reperfusion (I/R) injury (3). HO-1 knockout increases cardiac damage after I/R injury (4), whereas HO-1 overexpression decreases (5) cardiac damage after I/R injury. Thus, CO has numerous beneficial actions in the heart, vasculature, and other systems (6, 7), many of which are mediated by its actions on distinct ion channels (8, 9); however, despite the beneficial effects of endogenous CO, exposure to exogenous CO is hazardous. CO poisoning accounts for more than 50% of fatal poisonings (10–12) and a large-scale U.S. survey clearly established an association between ambient CO and increased risk of hospitalization as a result of cardiovascular complaints, including arrhythmias (13). This supports previous studies that have implicated environmental CO exposure in myocardial dysfunction (14, 15). Chronic, lower-level exposure to CO produces cardiac injury and fibrosis (16, 17), and acute environmental exposure can lead to arrhythmias and risk of associated sudden death (15, 18).

Ion channels are a major group of proteins that is modulated by CO, which occurs *via* numerous signaling pathways (8). In rat cardiac myocytes, we have demonstrated that CO promotes early afterdepolarization (EAD)–like arrhythmias that are reminiscent of long QT syndrome 3 (LQT3) arrhythmias by increasing the amplitude of the late Na^+^ current, *I*_NaL_ (19), an effect that is attributable to the activation of NO formation by nNOS (20) and subsequent S-nitrosylation of the channel. Other studies have shown that CO inhibits the cardiac l-type Ca^2+^ current (21) and inward rectifier K^+^ current (22), each by distinct mechanisms. These studies highlight the fact that the proarrhythmic effects of CO are complex and involve the regulation of multiple ion channels; however, their translational impact is limited because all studies to date have been conducted in rat cardiac myocytes, and there are major electrophysiologic differences between rat cardiac myocytes and those from larger mammals, including humans. In particular, the plateau phase of the ventricular action potential (AP) is brief or absent in rat myocytes (23, 24) compared with guinea pig myocytes. This presumably arises from differences in expression—and relative expression levels—of ion channels that contribute to the AP.

One key ion channel, ether-a-go-go–related gene (ERG) (Kv11.1), which is primarily responsible for myocyte repolarization (giving rise to the K^+^ current *I*_Kr_), seems to be expressed at low levels in rat tissue (23, 24) but is prominent in other species, such as guinea pig and human. The importance of Kv11.1 is reflected in the fact that numerous ERG mutations give rise to LQT2, one of the most common forms of long QT syndrome, which increases patients’ vulnerability to arrhythmias and sudden death (25–29). Furthermore, the U.S. Food and Drug Administration requires that all new drugs be tested for long QT-associated cardiac risk (30), as so many diverse drugs modulate this channel [reviewed previously (30–32)], which causes acquired long QT syndrome, a major safety challenge for the pharmaceutical industry. Here, we explore the ability of CO to modulate recombinant human (h)ERG channels and assess the potential arrhythmic impact of such modulation on ventricular myocytes from guinea pigs, which abundantly express Kv11.1 and display a prominent AP plateau phase that is reminiscent of human tissue.

## MATERIALS AND METHODS

### Isolation of guinea pig myocytes

Dunkin Hartley guinea pigs (300–350 g; Charles River UK, Kent, United Kingdom) were euthanized in accordance with UK Home Office Guidance on the Operation of Animals (Scientific Procedures) Act 1986 and institutional guidelines. Isolated hearts were perfused *via* the aorta with warm (37°C), oxygenated tyrode solution that contained (mM) 135 NaCl, 6 KCl, 0.33 NaH_2_PO_4_, 5 Na pyruvate, 1 MgCl_2_, 10 HEPES, and 10 glucose, adjusted to pH 7.4 with NaOH, for 5 min in the absence of Ca^2+^. To disaggregate cells, each heart was perfused with Ca^2+^-free tyrode solution that contained collagenase type II (100 U/ml; Worthington, Lorne, VIC, Australia), protease (0.66 mg/ml; Sigma-Aldrich, St. Louis, MO, USA), and bovine serum albumin (1.66 mg/ml; Sigma-Aldrich) for 10 min, then washed with 1 mM Ca^2+^-containing tyrode solution for 5 min. Ventricles were minced and gently shaken every 5 min in the latter solution. Cells were maintained in 2 mM Ca^2+^-containing tyrode solution.

### Expression of hERG in human embryonic kidney 293 cells

Human embryonic kidney 293 (HEK293) cell lines that stably express hERG1a (Kv11.1) were generated by using a pCEP4 plasmid that contained hERG cDNA and transfected by using a lipofectamine method (Thermo Fisher Scientific, Waltham, MA, USA). A hygromycin resistance gene was used for the selection of stable lines. Single colonies were picked and examined for hERG currents by using whole-cell patch-clamp recordings (see below). Positive clones were cultured in minimum essential medium (Thermo Fisher Scientific) that was supplemented with fetal bovine serum (10%), nonessential amino acids (1%), an antibiotic antimycotic mix (1%), glutamax (1%; Thermo Fisher Scientific), and hygromycin (100 µg/ml; Calbiochem, San Diego, CA, USA). C723S mutation was introduced using the QuikChange site-directed mutagenesis kit (Stratagene, Cambridge, United Kingdom) according to manufacturer instructions. All constructs were verified by DNA sequence analysis.

### Electrophysiology—recombinant hERG

Coverslip fragments with attached cells were transferred to a perfused (3–5 ml/min) recording chamber that was mounted on an Olympus CK40 inverted microscope (Olympus, Tokyo, Japan). The chamber (80–100 μl volume) was perfused at room temperature (21–23°C) with a solution that was composed of (mM) 140 NaCl, 4 KCl, 1.8 CaCl_2_, 1 MgCl_2_, 10 HEPES, and 10 d-glucose (pH adjusted to 7.4 with NaOH). Whole-cell patch-clamp recordings were made by using patch pipettes of 4- to 7-MΩ resistance when filled with the intracellular solution that was composed of (mM) 110 KCl, 10 NaCl, 10 EGTA, 1 MgCl_2_, 10 HEPES, and 5 MgATP (pH 7.2, KOH). Series resistance was compensated for by 60–90% and hERG currents were fully activated by using a 1-s prepulse to +40 mV followed by depolarizing steps that ranged from −100 to +40 mV applied in 10-mV increments for 3 s (holding potential of −80 mV; Fig. 2*A*). Currents were measured at the end of the 3-s test pulse without any corrections for residual voltage errors. For time series plots (*e.g.*, Fig. 2*D*), hERG tail currents were repeatedly activated by using a 2-s step depolarization to +40 mV applied from a holding potential of −80 mV, followed by a 2-s pulse to −40 mV to record tail currents, before repolarization to −80 mV. All protocols were applied at 0.1 Hz.

### Electrophysiology—cardiac myocytes

Myocytes were perfused after settling in the perfusion chamber. Perfusion and pipette composition was exactly as used in HEK293 cell recordings. Myocytes were voltage clamped at −40 mV and step depolarized to +20 mV for 750 ms before repolarization to −40 mV. *I*_Kr_—that is, the outward K^+^ current passing through ERG channels—was evident as a tail current observed after repolarization (33–35). APs were evoked in current-clamp mode (*I* = 0) by applying supramaximal depolarizing current injections (5-ms duration, 0.1 Hz). AP duration (APD) at 20, 50, and 90% of maximal amplitude was measured. Percentage change was calculated as follows: (APD in the presence of drug/control APD) × 100. Statistical analysis was performed by using paired Student’s *t* tests, where *P* < 0.05 was considered significant.

### Peroxynitrite detection

Attached cells on coverslips were incubated with 2-[6-(4′-amino) phenoxy-3H-xanthen-3-on-9-yl] benzoic acid (APF; 10 µM) that was dissolved in HEPES-buffered saline for 1 h at 37°C in the dark. Coverslip fragments were placed on a glass slide that contained 200 µl HEPES-buffered saline with 10 µM APF. Changes in fluorescence intensity were measured over 10 min by using a Zeiss laser scanning confocal microscope (LSM 510; Zeiss, Oberkochen, Germany). APF was excited at 488 nm, emission was monitored at 510 nm, and images were obtained by using Zeiss AIM software. Identical settings were used for each test condition. For APF experiments that involved L-NG-nitroarginine methyl ester (L-NAME), cells were incubated with both 10 µM APF and 1 mM L-NAME for 1 h at 37°C before recording. Experiments that involved FeTPPS [5,10,15,20-tetrakis-(4-sulfonatophenyl)-porphyrinato-iron(III)] used cells that were incubated with both 10 μM APF and 25 μM FeTPPS for 1 h at 37°C.

## RESULTS

**Figure 1.**
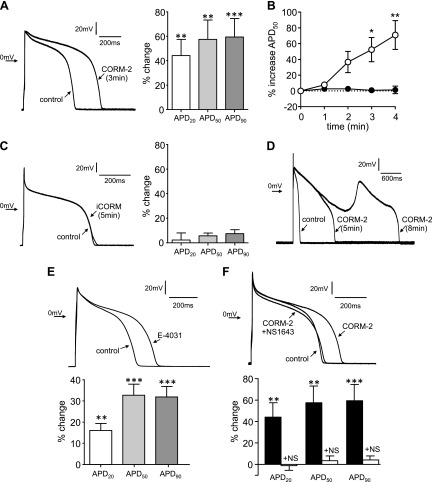
CO induces EAD-like arrhythmias in guinea pig myocytes. *A*) Example APs evoked in a guinea pig myocytes before (control) and after 3 min of exposure to the CO donor, CORM-2 (10 μM; left). Mean ±sem percentage changes in APD caused by 10 μM CORM-2 after 3 min (*n* = 14; right). *B*) Time course plot of the increase in APD_50_ caused by 10 μM CORM-2 (open symbols, *n* = 14) and 10 μM iCORM (solid symbols; *n* = 5). Each point is the mean ±sem percentage change in APD_50_ duration. *C*) Example APs evoked in a guinea pig myocyte before (control) and after 5 min of exposure to the iCORM (10 μM; left). Mean ±sem percentage changes in APD caused by 10 μM iCORM after 5 min (*n* = 5; right). *D*) Examples of evoked APs during more prolonged exposure to CORM-2. Note the emergence of EAD-like AP. *E*) Example APs evoked in a myocyte before (control) and after 4 min of exposure to E-4031 (1 μM; top). Mean ± sem percentage changes in APD caused by 1 μM E-4031 after 5 min (*n* = 6; bottom). *F*) Example APs evoked in a myocyte before (control) and after 3 min of exposure to CORM-2 (10 μM), then after 5 min exposure to NS1643 (1 µM) in the continued presence of CORM-2 (top). Mean ±sem percentage changes in APD caused by 10 μM CORM-2 alone (solid bars) and in the presence of 1 µM NS1643 (*n* = 6; bottom). **P < 0.01; ***P < 0.001, determined by paired Student’s *t* tests of control (predrug) values and those observed in the presence of drugs applied to the same cells.

**Figure 1*A*** shows representative APs that were evoked in a guinea pig myocyte before and during bath application of the CO donor, CO-releasing molecule 2 (CORM-2; 10 μM). In the presence of CORM-2, APD gradually increased as quantified at 3 min in Fig. 1*A*. The time course of increase is shown in Fig. 1*B* for APD_50_. Also plotted in Fig. 1*B* (example in Fig. 1*C*) is the lack of effect of the inactive compound, inactive CORM (iCORM). In all 11 myocytes in which the effects of CORM-2 were followed for >5 min, EAD arrhythmias were observed (Fig. 1*D*). Qualitatively similar increases in APD were observed during exposure to the ERG inhibitor, E-4031 (1 μM; Fig. 1*E*). Furthermore, the effect of CORM-2 to prolong APD was fully reversed by the ERG activator, NS1643 (Fig. 1*F*). Collectively, these findings suggest that the proarrhythmic effects of CO in guinea pig myocytes may arise as a result of the inhibition of ERG channels.

**Figure 2.**
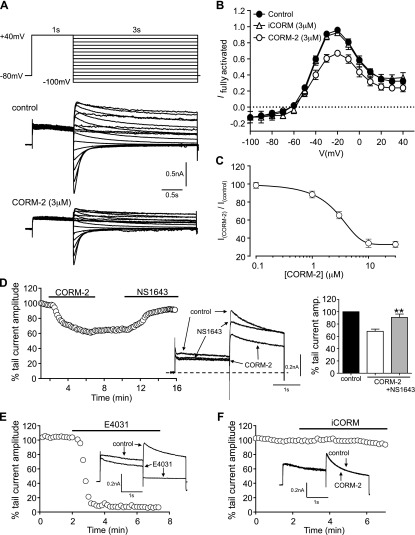
CO inhibits recombinant hERG. *A*) Illustration of the voltage-clamp protocol used to generate the fully activated current-voltage relationships in HEK293 cells (top). Example families of currents evoked by this protocol in HEK293 cells that stably express hERG before (control) and after (CORM-2) exposure of cells to 3 μM CORM-2 (bottom). *B*) Mean ± sem current voltage-relationships obtained from cells before (solid circles, *n* = 9) and during exposure to CORM-2 (3 μM; open circles, *n* = 9) or iCORM (3 μM; open triangles, *n* = 8). *C*) Concentration-response relationship obtained by determining the percentage inhibition of the hERG tail current caused by CORM-2 as determined by using a 2-step pulse protocol of 2 s of depolarization to +40 mV followed by a 2-s step to −40 mV from a holding potential of −80 mV and applied every 10 s. Each point plotted is the mean ± sem taken from between 3 and 6 cells. *D*) Time-series plot in which normalized peak tail current amplitudes, evoked by successive step depolarizations as indicated in panel *C*, are plotted against time (left). For the periods indicated by the horizontal bars, cell was exposed to 3 μM CORM-2 or 3 µM NS1643, as indicated. Superimposed example traces from the same experiment that illustrate the effects of CORM-2 and NS1643, as indicated (center). Mean ± sem (*n* = 5) percentage inhibition of the peak tail current caused by 3 µM CORM-2 alone (white bar) and reversal of this inhibition by 3 μM NS1643 (gray bar; right). **P < 0.01, significant reversal of CORM-2–inhibited current amplitudes by NS1643 (comparing white and gray bars). *E*) Time-series plot evoked as in panel *D*, but in this case the cell was exposed to 3 μM E-4031. Superimposed example traces from the same experiment that illustrate the effect of E-4031, as indicated (inset). *F*) Same as panel *E*, except that the cell was exposed to 3 μM iCORM.

To explore the modulation of ERG currents by CO in isolation without contamination from other native currents in myocytes, we stably expressed the human isoform (hERG) in HEK293 cells. Depolarization evoked robust outward currents, with tail currents evoked by repolarization to different potentials, as illustrated in **Fig. 2*A***. At more positive repolarization values, tail currents were characteristically larger than those that were observed during depolarizing pulses as a result of the relief of rapid inactivation that occurs before deactivation (Fig. 2*A*). Currents at all APs were reduced in amplitude by 3 μM CORM-2 (Fig. 2*A, B*), and after determining a concentration-response relationship (Fig. 2*C*), this concentration was used for additional experiments.

Note the lack of effect for 3 μM iCORM (Fig. 2*B*). Unless otherwise stated, we then employed a protocol of step depolarizations that were applied repeatedly from −80 to +40 mV for 2 s, followed by repolarization to −40 mV. Resultant tail currents were measured at their peak. This protocol highlighted that inhibition by CORM-2 was essentially irreversible over periods that ranged from 3 to 5 min (Fig. 2*D*); however, current amplitudes were recovered by the ERG activator, NS1643 (Fig. 2*D, E*). Currents were also strongly inhibited by E-4031 (Fig. 2*E*; 3 µM caused 75 ± 5.5% inhibition; *n* = 4; *P* < 0.001) far more rapidly than by CORM-2 (compare Fig. 2*D, E*). Currents that were evoked by repeated step depolarizations were essentially unaffected by iCORM (Fig. 2*F*, representative of 6 cells).

**Figure 3.**
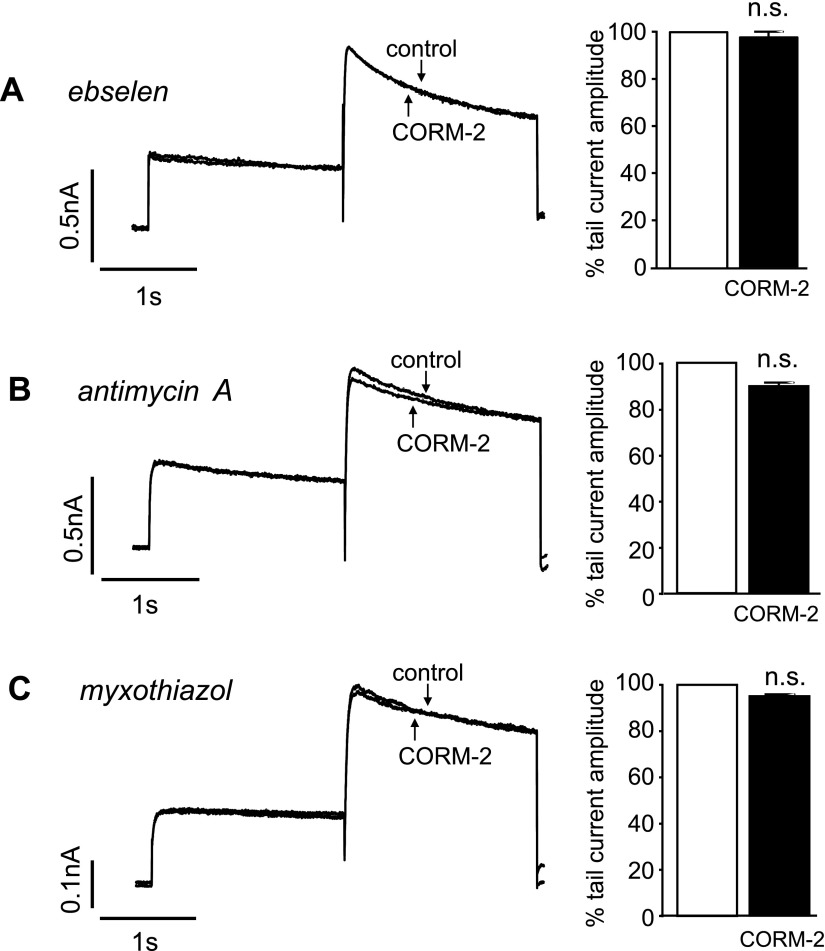
CO inhibition of hERG involves mitochondrial ROS. *A*) Superimposed example currents from the same cell that illustrate the lack of effect CORM-2 (3 µM) after pretreatment of the cell with 100 nM ebselen for 30 min (left). Mean ± sem (*n* = 5) percentage inhibition of the peak tail current caused by 3 µM CORM-2 after pretreatment with 100 nM ebselen (right). *B*, *C*) Same as panel *A*, except that cells were pretreated with either antimycin A (*B*; *n* = 5) or myxothiazol (*C*; *n* = 6), both at 1 μM for 1 h at 37°C. N.s., not significant compared with paired *t* tests of amplitudes before and during CORM-2 application.

CO regulates ion channels *via* numerous mechanisms (15). The ability of CORM-2 to inhibit recombinant hERG was blocked by the antioxidant ebselen (100 nM; **Fig. 3*A***), which indicates the likely involvement of reactive oxygen species (ROS). Mitochondria represent a major source of ROS that can be increased by CO (18). To investigate their involvement, we examined the effects of 2 mitochondrial inhibitors. Pretreatment of cells with either antimycin A (Fig. 3*B*) or myxothiazol (Fig. 3*C*)—both applied at 1 μM for 1 h at 37°C—almost fully prevented the inhibition of hERG by CORM-2, which suggests that CO-mediated inhibition of hERG involves mitochondria-derived ROS.

**Figure 4.**
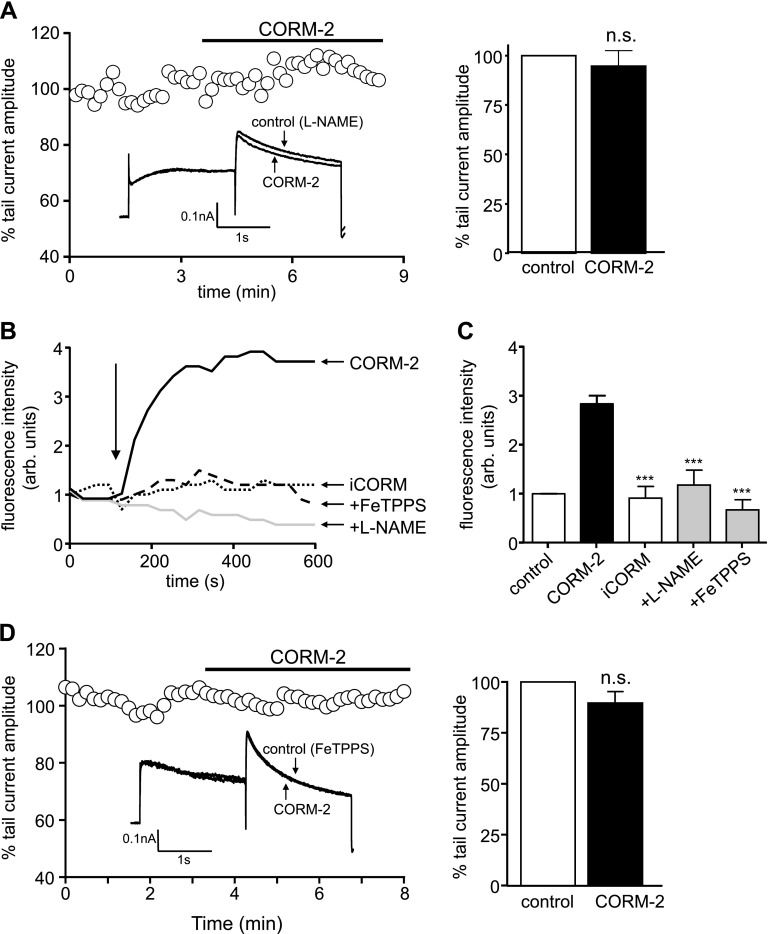
CO inhibition of hERG involves the formation of peroxynitrite. *A*) Time-series plot in which normalized peak tail current amplitudes, evoked by step depolarizations from −80 to +40 mV followed by a repolarization to −40 mV, are plotted against time. For the period indicated by the horizontal bar, the cell was exposed to 3 μM CORM-2, as indicated. Before this recording, the cell was pretreated with 1 mM L-NAME for 1 h at 37°C. Superimposed example currents from the same cell that illustrate the lack of effect CORM-2 (3 µM) after pretreatment of the cell with L-NAME (inset). Mean ± sem (*n* = 7) percentage inhibition of the peak tail current caused by 3 µM CORM-2 after pretreatment with 1 mM L-NAME (right). *B*) Example recordings of APF fluorescence monitored in HEK293 cells that stably express hERG. At the point indicated by the arrow, cells were exposed to CORM-2, iCORM, and CORM-2 after pretreatment with 25 μM FeTPPS for 1 h at 37°C or CORM-2 after pretreatment with 1 mM L-NAME for 1 h at 37°C, as indicated. *C*) Bar graph plotting mean ± sem (determined from 5 or 6 recordings in each case) APF fluorescence determined at room temperature after exposure of cells to CORM-2, iCORM, or CORM-2 in FeTPPS- or L-NAME–pretreated cells. Control (open bar) represents time-matched recordings during which no drugs were added. ***P < 0.001, unpaired Student’s *t* test comparisons *vs.* effects of CORM-2. *D*) Time-series plot as in panel *A*, except that the cell was pretreated with 25 μM FeTPPS 1 h. Superimposed example currents from the same cell that illustrate the lack of effect CORM-2 (3 µM) after pretreatment of the cell with FeTPPS (inset). Mean ± sem (*n* = 6) percentage inhibition of the peak tail current caused by 3 µM CORM-2 after pretreatment with 25 µM FeTPPS (right). n.s., not significant compared with paired Student’s *t* test of amplitudes before and during CORM-2 application.

**Figure 5.**
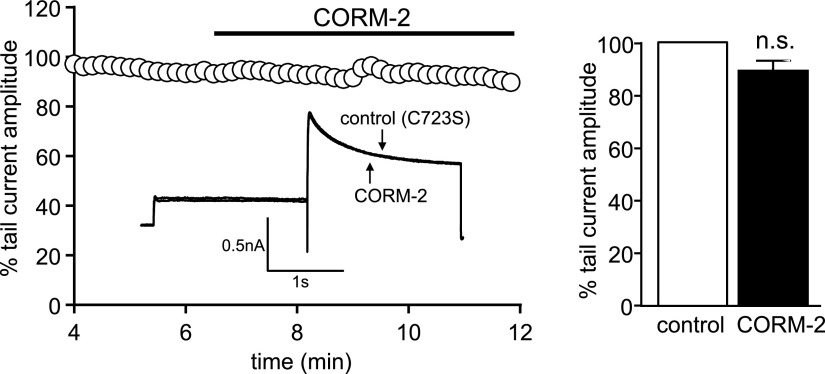
hERG C723S mutant is insensitive to CO. Time-series plot in which normalized peak tail current amplitudes, evoked by successive step depolarizations from −80 to +40 mV followed by a repolarization to −40 mV, are plotted against time. For the period indicated by the horizontal bar, the cell was exposed to 3 μM CORM-2, as indicated. This recording was obtained from a HEK293 cell that was stably transfected with a C723S mutant form of hERG. Superimposed example currents from the same cell that illustrate the lack of effect CORM-2 (3 µM) on the C723S mutant form of the channel (inset). Mean ± sem (*n* = 5) percentage inhibition of the peak tail current caused by 3 µM CORM-2 in cells that express the C723S mutant form of hERG (right). N.s., not significant compared with paired *t* test of amplitudes before and during CORM-2 application.

We also investigated the role of NO in CO inhibition of hERG, as NO mediates increases in *I*_NaL_ by CO in rat cardiac myocytes (16). Pretreating cells with 1 mM L-NAME (1 h, 37°C) to prevent NO formation abolished the inhibitory effects of CORM-2 (**Fig. 4*A***), which indicates that CO inhibition of hERG requires NO formation. The dependence of CO inhibition of hERG on both ROS and NO raised the possibility that CO stimulates formation of peroxynitrite (ONOO^−^); therefore, we investigated whether CO could generate detectable levels of ONOO^−^ by using the ONOO^−^-sensitive fluoroprobe, APF. As shown in Fig. 4*B*, CORM-2, but not iCORM, evoked an increase in APF fluorescence, which was indicative of ONOO^−^ formation that was abolished by pretreatment of cells with L-NAME, as well as by the ONOO^−^ scavenger, FeTPPS [5,10,15,20-tetrakis-(4-sulfonatophenyl)-porphyrinato-iron(III); 25 μM], which converts ONOO^−^ to nitrate (36). Mean APF data are plotted in Fig. 4*C*. Pretreatment of cells with FeTPPS (1 h at 37°C, 25 μM) also prevented the inhibition of hERG by CORM-2 (Fig. 4*D*). Our findings strongly suggest that CO inhibited hERG *via* ONOO^−^-mediated oxidation. Previous studies have shown that cysteine 723 is a C-terminal residue that confers sensitivity to oxidants (37). We generated a C723S mutant to explore the involvement of this residue in CO sensitivity of hERG. As seen in Fig. 4*D*, this mutant form of hERG was insensitive to CORM-2 (**Fig. 5**).

**Figure 6.**
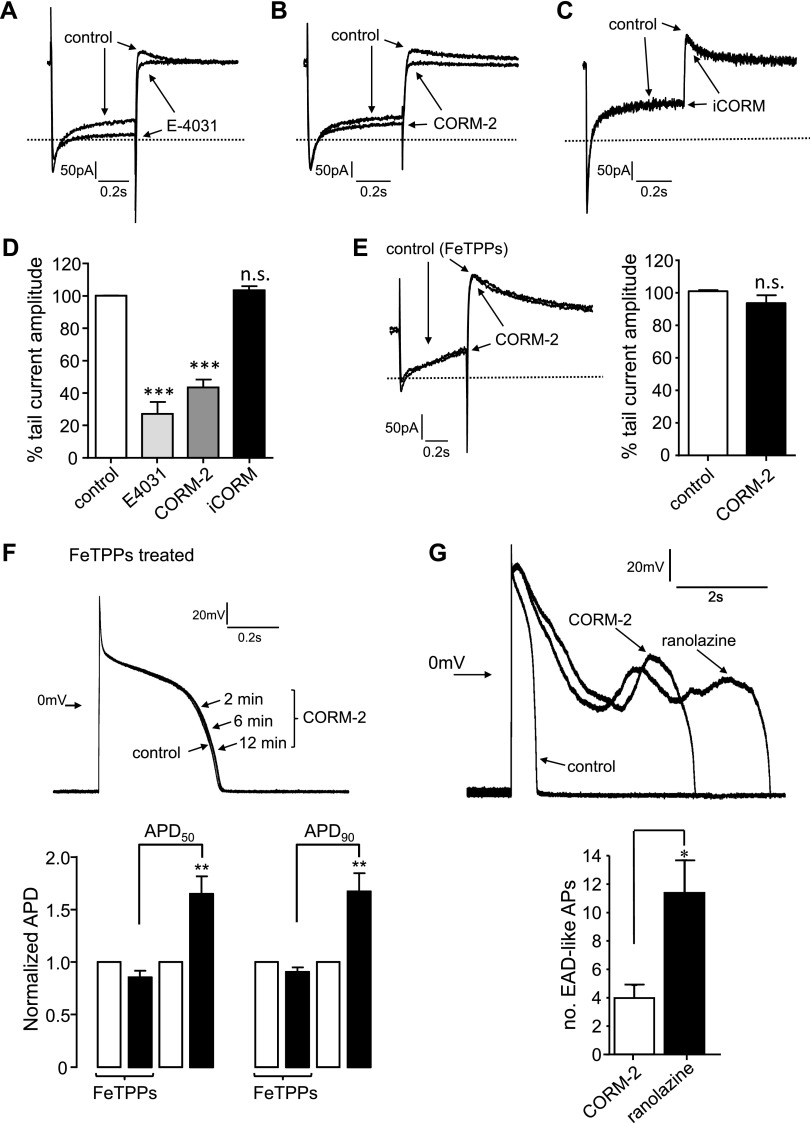
CO inhibits native ERG in guinea pig myocytes. *A*) Example currents in a guinea pig ventricular myocyte evoked by step depolarization from −40 to +20 mV, then repolarization to −40 mV. The current attributable to ERG channels is the transient outward current observed on repolarization. The horizontal line in all traces of this figure (*A*–*C*, *E*) indicate 0 current level. Two superimposed recordings are shown: before and during exposure of the myocyte to 1 µM E-4031. *B*, *C*). Same as panel *A*, except that cells were exposed either to CORM-2 (10 µM; *B*) or iCORM (10 µM; *C*). *D*) Bar graph plotting mean ± sem (*n* = 5–11 in each case) percentage inhibition of the peak tail current amplitude caused by application of E-4031, CORM-2, and iCORM (as exemplified in panels *A*–*C*). ***P < 0.001, paired Student’s *t* tests of control *vs.* drug treatments. *E*) Same as panel *B*, except the cell was pretreated for 1 h at 37°C with 25 µM FeTPPS (left). Mean ± sem (*n* = 9) percentage inhibition of the peak tail current caused by 10 µM CORM-2 in cells that were pretreated for 1 h at 37°C with 25 μM FeTPPS (right). *F*) Example APs evoked in a guinea pig myocyte previously pretreated for 1 h at 37°C with 25 μM FeTPPS before (control) and during exposure to 10 µM CORM-2 for the times indicated (top). Bar graphs plotting normalized APD_50_ and APD_90_ values before (open bars) and during (solid bars) exposure to 10 µM CORM-2 (bottom). Cells were either pretreated with 25 μM FeTPPS for 1 h (as indicated) or did not receive pretreatment. **P < 0.01 compared with paired *t* tests of APDs before and during CORM-2 application. *G*) Example APs recorded in a guinea pig myocyte before drug exposure (control), after 9 min of exposure to 10 µM CORM-2, and after 5 min of exposure to 20 µM ranolazine, which was applied immediately after CORM-2 (top). Bar graph plotting mean ± sem (*n* = 5) number of EAD-like APs recorded in myocytes over a period of 10 min during CORM-2 exposure, and during ranolazine exposure, as indicated (bottom). N.s., not significant. *P < 0.05, unpaired Student’s *t* test.

Collectively, our findings suggest that CO inhibition of ERG (Kv11.1) can account for the proarrhythmic effects in guinea pig myocytes. To further investigate this, we examined the ability of CORM-2 to inhibit *I*_Kr_ (corresponding to Kv11.1) in guinea pig myocytes. Following established protocols (35), currents were obtained by using depolarizing steps to +40 mV for 750 ms, followed by a repolarization to −40 mV from a holding potential of −80 mV. These outward currents were markedly reduced by 1 µM E-4031 (**Fig. 6*A, D***). Similarly, CORM-2 (10 μM) reduced these outward currents (Fig. 6*B, D*); however, iCORM (10 μM) was without effect (Fig. 6*C, D*). Of importance, pre-exposure (30 min, 37°C) of guinea pig myocytes to FeTPPs (25 μM) abolished the subsequent effects of 10 μM CORM-2 on transient outward currents (Fig. 6*E*). Thus, native ERG currents in myocytes seemed to be inhibited by CO *via* the same mechanism as that observed for recombinant hERG—that is, *via* the formation of ONOO^−^.

We also observed that the effects of CORM-2 on APD were abolished by FeTPPs (Fig. 6*F*). Finally, ranolazine (20 μM), an inhibitor of *I*_NaL_ that reverses the arrhythmic effects of CO in rat myocytes (19), did not prevent the proarrhythmic effects of CO in guinea pig myocytes. In these experiments, APs were evoked, and CORM-2 was applied for a period of 10 min. During this period, the number of clear EAD-like APs (as exemplified in Fig. 6*G*) was averaged (see bar graph). Then, the perfusate was switched to one that contained ranolazine (20 µM). Over the following 10 min, the incidence of EAD-like APs significantly increased (Fig. 6*G*), which suggests that modulation of ERG (Kv11.1) is the dominant mechanism that underlies the proarrhythmic effects of CO in guinea pig myocytes.

## DISCUSSION

The present study demonstrates that inhibition of ERG K^+^ channels is the dominant proarrhythmic effect of CO in guinea pig myocytes. Results are consistent with previous studies that have reported that CO prolongs ventricular APD (19, 22), but the underlying mechanisms are strikingly different. Previously, reports have indicated that CO increased *I*_NaL_ (19) or inhibited inward rectifier K^+^ currents (22) to prolong APD, but these studies used rat ventricular myocytes in which the activity of ERG channels is negligible (24, 38). Rat and mouse myocytes are valuable, the latter particularly for transgenic studies, but their brief APD—and channel expression profile—compared with human cardiac APD, limits their translational value. By contrast, guinea pig myocyte APs display a significant plateau phase that is reminiscent of human ventricular tissue (39) and express functionally important ERG K^+^ channels that are comparable to hERG (35).

Clearly, multiple signaling pathways mediate the CO regulation of ion channels in cardiac myocytes: l-type Ca^2+^ channels are inhibited by CO *via* mitochondrial ROS formation (21), whereas augmentation of *I*_NaL_ by CO required NO formation (19). In guinea pig myocytes, CO increases both NO (Fig. 4) and mitochondrial ROS production (Fig. 3), which form ONOO^−^ to inhibit ERG channels *via* oxidation of C723 (Fig. 5). Liang *et al.* (22) indicated that CO inhibits inward rectifier K^+^ channels by disrupting their interaction with phosphatidylinositol (4, 5)-bisphosphate, although the underlying signaling pathway was not explored. Thus, it seems that CO can regulate multiple cardiac myocyte ion channels *via* different signaling pathways, and that each of these could individually contribute to the remodeling of the AP shape and duration. The present study does not discount any of these alternate pathways or ion channel targets as contributory factors to the overall effect observed in guinea pig myocytes, but suggests strongly that CO inhibition of Kv11.1 is of major importance.

In rats, *I*_NaL_ augmentation by CO is central to its proarrhythmic effects, the reversal of which was achieved by ranolazine, an inhibitor of *I*_NaL_. The present study suggests that augmentation of *I*_NaL_ does not contribute significantly to APD prolongation and EAD-like arrhythmias in guinea pig myocytes, as these were exacerbated and not reversed by ranolazine (Fig. 5). Exacerbation, although not studied further, is consistent with earlier work (40) and may be attributable to the inhibition of ERG channels by ranolazine (41, 42). Furthermore, FeTPPS prevented the arrhythmic activity and inhibition of ERG channels by CO in guinea pig myocytes (Fig. 5), but augmentation of *I*_NaL_ by CO in rats occurred independently of ROS formation (19). These findings do not preclude the idea that CO can induce ONOO^−^ formation in rat cardiac myocytes. Instead, they suggest that ERG channels seem to be particularly sensitive to ONOO^−^, an effect that is undetectable in rats because of their extremely low expression. It is conceivable that colocalization of mitochondria (the source of ROS), NOS, and ERG channels favors localized ONOO^−^ formation, which is sufficiently close to ERG channels to cause their selective inhibition. Alternatively, ERG channels might discriminate between different ROS. We consider this highly unlikely, as hERG can be modulated by ROS generated by various experimental means (37, 43).

Currently, it is unclear how to reconcile the proarrhythmic effects of CO described here and elsewhere (19) with the reported cardioprotective effects of HO-1 induction, many of which are attributable to CO (9). Cardiac I/R injury is worsened in HO-1^+/−^ mice (44), whereas HO-1 overexpression provides protection (5), and this protective effect was also observed after the administration of the water-soluble CO donor, CORM-3 (3, 45). Indeed, CORM-3 has a positive inotropic effect on the isolated rat heart (46). This effect, which was not mimicked by a more slowly acting CO donor, was attributable to the activation of cyclic GMP and could also be abolished by the inhibition of Na^+^/H^+^ exchange. Neither mechanism is involved in the modulation of ERG—or other relevant channels—in myocytes. Details of APs were not investigated. These cardioprotective effects of CO suggest that it may have therapeutic potential; however, it is clear from the present study that the current understanding of how CO affects cardiac excitability and function is far from complete, particularly as rat tissue has been the chief source of information to date. Our findings suggest that interventions that target/restore ERG activity may provide a novel and effective approach to treating CO-induced arrhythmias in humans.

## Abbreviations

AP: action potential
APD: action potential duration
APF: 2-[6-(4′-amino)phenoxy-3H-xanthen-3-on-9-yl] benzoic acid
CORM-2: CO-releasing molecule
EAD: early afterdepolarization
ERG: ether-a-go-go–related gene
FeTPPS: 5,10,15,20-tetrakis-(4-sulfonatophenyl)-porphyrinato-iron(III)
HEK293: human embryonic kidney 293
HO: heme oxygenase
iCORM: inactive CO-releasing molecule
I/R: ischemia/reperfusion
L-NAME: L-NG-nitroarginine methyl ester
LQT2/3: long QT syndrome 2/3
ROS: reactive oxygen species
